# Prevented Sudden Cardiac Death and Neurologic Recovery in Inherited Heart Diseases

**DOI:** 10.3389/fcvm.2021.634300

**Published:** 2021-03-15

**Authors:** Juan P. Hernández del Rincón, Mari C. Olmo Conesa, Ana Rodríguez Serrano, Helena García Pulgar, David López Cuenca, Carmen Muñoz Esparza, Marina Navarro Peñalver, Juan José Santos Mateo, Elisa Nicolás Rocamora, Cristina Gil Ortuño, María Sabater-Molina, Juan Ramón Gimeno Blanes, Francisco Pastor Quirante

**Affiliations:** ^1^Instituto de Medicina Legal y Ciencias Forenses, Murcia, Spain; ^2^Departamento de Medicina Legal, Universidad de Murcia, Murcia, Spain; ^3^Instituto Murciano, de Investigación Biosanitaria (IMIB), Murcia, Spain; ^4^Unidad de Cardiopatías Hereditarias, Servicio de Cardiología, Hospital Universitario Virgen de la Arrixaca, Murcia, Spain; ^5^Departamento de Medicina Interna, Universidad de Murcia, Murcia, Spain; ^6^European Reference Networks (Guard-Heart), Amsterdam, Netherlands; ^7^Red de investigación Cardiovascular (CIBERCV), Instituto de Salud Carlos III, Madrid, Spain; ^8^Servicio de Anatomía Patológica, Hospital General Universitario Reina Sofía, Murcia, Spain; ^9^Departamento de Anatomia Patologica, Universidad de Murcia, Murcia, Spain

**Keywords:** sudden cardiac death, cardiomyopathy, channelopathy, inherited cardiovascular diseases, prevented sudden cardiac death

## Abstract

**Introduction:** Inherited cardiovascular diseases are an important cause of sudden cardiac death (SD). The use of risk scores identify high risk patients who would benefit from an implantable cardioverter-defibrillators (ICDs). The development of automated devices for out-of-hospital cardiac arrest improves early resuscitation. The objective of the study is to quantify prevented SD and the neurological recovery of patients with inherited cardiovascular diseases.

**Methods:** Two hundred fifty-seven cases of SD (age 42 ± 18 years, 79.4% men) of non-ischemic cardiac cause were prospectively collected during the study period (2009–17). Fifty three (20.6%) had a resuscitated cardiac arrest (RCA) (age 40 ± 18 years, 64.2% male). Epidemiological, clinical and autopsy aspects were analyzed. Prevented SD was defined as a combination of RCA and appropriate ICD therapy cases.

**Results:** An autopsy was performed in 157/204 (77.0%) cases who died. There were 19 (12.1%) cases with a negative autopsy. The diagnosis of cardiomyopathy and channelopathy was 58.0 and 18.7%, respectively. Female sex and confirmed or suspected channelopathy were associated with successful resuscitation. The percentage of prevented SD remained low during the study period (mean 35.6%). 60.4% of RCA cases presented good neurological outcome. There was no association between neurological recovery and therapeutic hypothermia, but there was association with time of resuscitation (min).

**Conclusion:** A fifth part of non-ischemic cardiac arrests were resuscitated. Female sex and channelopathies were more prevalent among RCA. Two thirds of RCA had a good neurological recovery.

## Introduction

Sudden death (SD) is the unexpected, sudden and natural death of a previously health or clinically stable person (1). It is a devastating event, both for the family and the society, which affects people of all ages. SD can be the first manifestation of a heart disease, such as a cardiomyopathy or a channelopathy (2–4). The overall survival rate for cardiac arrest is about 10% (5, 6). The success rate increases in trained populations and in places equipped with automated external defibrillators. A family study facilitates early diagnosis of inherited heart diseases and risk stratification. Implantation of implantable cardioverter-defibrillators (ICD) allows for the effective treatment of malignant ventricular arrhythmias (5, 6).

The main objective of this study was to describe the most prevalent causes of both resuscitated and non-resuscitated non-ischemic SD in our environment. The secondary objectives were: (1) to assess the impact of cardiac arrest prevention strategies over time and (2) to analyze the extent of neurologic recovery in cases of resuscitated cardiac arrest (RCA).

## Materials and Methods

### Design and population

**Figure 1:**
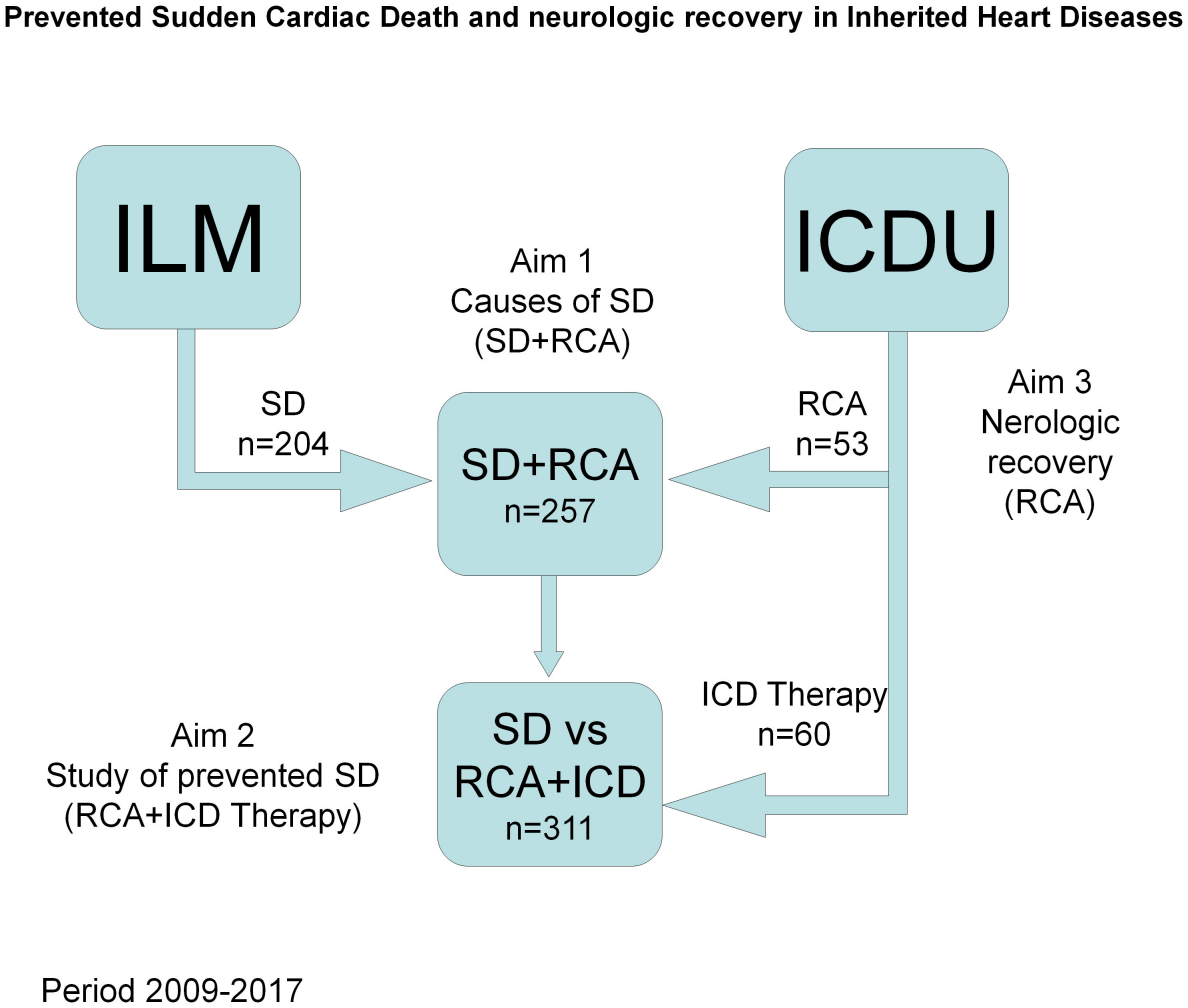
Study design. Selection of population and objectives. ILM, Institute for Legal Medicine and Forensic Sciences; ICDU, Inherted Cardiac Disease Unit; SD, Sudden death; RCA, Resuscitated Cardiac Arrest.

Two fifty-seven individuals (age 41.9 ± 18.3 years, 204, 79.4% male) with non-ischemic cardiac-related SD were included in this prospective study. The patients were assessed between January 2009 and December 2017 at the Institute for Legal Medicine (204 (79.4%) cases, age 42.3 ± 16.4 years, 170 (83.3%) male) or at the different intensive care units (53 (20.6%) resuscitated patients, age 40.2 ± 24.2 years, 34 (64.2%) male) in the Region of Murcia (Figure 1 and Supplementary Figure 1). The total mean reference population for the observation period was 1,466,032 inhabitants.

Cases were prospectively evaluated and data included in a specific database. All cases had a final or suspected diagnosis of cardiomyopathy or channelopathy (7–9). The evolution of the neurologic situation was analyzed retrospectively based on the available records and interview of patients and relatives. An informed consent was obtained from each patient or relatives and the study protocol conforms to the ethical guidelines of the 1975 Declaration of Helsinki as reflected in a priori approval by the institution's human research committee from University Hospital Virgen Arrixaca.

Genetic study was performed as part of the postmortem examination in cases with available sample. Pathogenicity study of variants was performed at our local Cardiogenetic Laboratory based on ACMG criteria (10). Following recommendations from guidelines (8, 11) first degree relatives (with or without positive genetic testing) were offered cardiac evaluation. Relatives from patients with pathogenic variants (according to ACMG criteria) were offered predictive test.

### Definitions

We included deceased patients whose death occurred within an hour after the onset of symptoms, or, in the absence of witnesses, when the deceased had been seen in good health at least 24 h before (1, 9). Cases with an extra-cardiac cause or whose origin was ischemic, valvular or attributed to toxic substance consumption were excluded. Individuals who had had a cardiac arrest and presented with spontaneous circulation recovery after resuscitation maneuvers were included as RCA.

In order to classify SD syndromes whose autopsy did not provide with a diagnosis, the accepted definitions were followed (12). SADS (Sudden Arrhythmic Death Syndrome): sudden death of individuals older than one with a full autopsy which could not establish the cause of death, but it was suspected to be arrhythmia. SUDS (Sudden Unexplained Death Syndrome): sudden death of individuals older than 1 year old without an autopsy or with an incomplete autopsy, where the cause of death could not be established. SIDS (Sudden Infant Death Syndrome): sudden death of an infant younger than one with a full autopsy which could not establish the cause of death, but it was suspected to be arrhythmia. SUDI (Sudden Unexplained Death in Infancy): sudden death of an infant younger than one without an autopsy or with an incomplete autopsy, where the cause of death could not be explained.

Prevented SD was defined as the sum of cases of RCA (*n* = 53) and cases with an appropriate ICD therapy (*n* = 60). Therefore, we included all patients diagnosed with a familial heart disease and with an ICD for primary prevention who had had a therapy for malignant ventricular arrhythmia at least once during the period of the study (8, 9). This later group consisted of 60 patients [age 48.8 ± 17.1 years, 48 (80.0%) male].

A “neurological damage” scale was defined. Good neurological state: full recovery, without significant limitations to lead a normal life. Moderate neurological impairment: there is impairment, but not enough to prevent the patient from performing the basic activities of daily life independently. Patients may present with speech, understanding and/or mobility impairment. Severe neurological impairment: patients are conscious, but with a high degree of impairment, which makes them dependent. Coma or vegetative state: patients are unconscious, presenting with severe neurological impairment, and are completely dependent. Exitus due to brain death.

### Statistical Analysis

The project was approved by the ethics committee of the coordinating center (Seneca project 2009). Clinical data were collected anonymously in a specific database. Statistical analysis was performed with SPSS (Statistical Package for the Social Sciences, for Windows, version 15.0).

To test hypotheses of qualitative variables, bivariate analysis was used with χ2 test. Student's *T*-test for independent samples allowed for testing hypotheses between one quantitative variable and one qualitative dichotomous variable, always previously using Levene's test to assess the equality of variances for appropriate interpretation. Logistic regression analysis was used to test for predictors of neurological recovery. The statistical significance established for the interpretation of results was *p* < 0.05.

## Results

### Characterization of Population

Of the total of 257 cardiac arrest cases included, 204 (79.4%) died (SD) and 53 (20.6%) were resuscitated (RCA). The mean incidence of non-ischemic SD in the Region of Murcia was 1.95 (CI 95%: 1.72–2.20) cases in every 100,000 inhabitants per year during the study period.

An autopsy was performed in 157/204 (77.0%) cases who died. In 47/204 (23.0%) cases, an autopsy was not performed or it was impossible to access the report. These cases were classified as SUDS based on personal and family history, among which there were 4 cases of SUDI.

**Figure 2:**
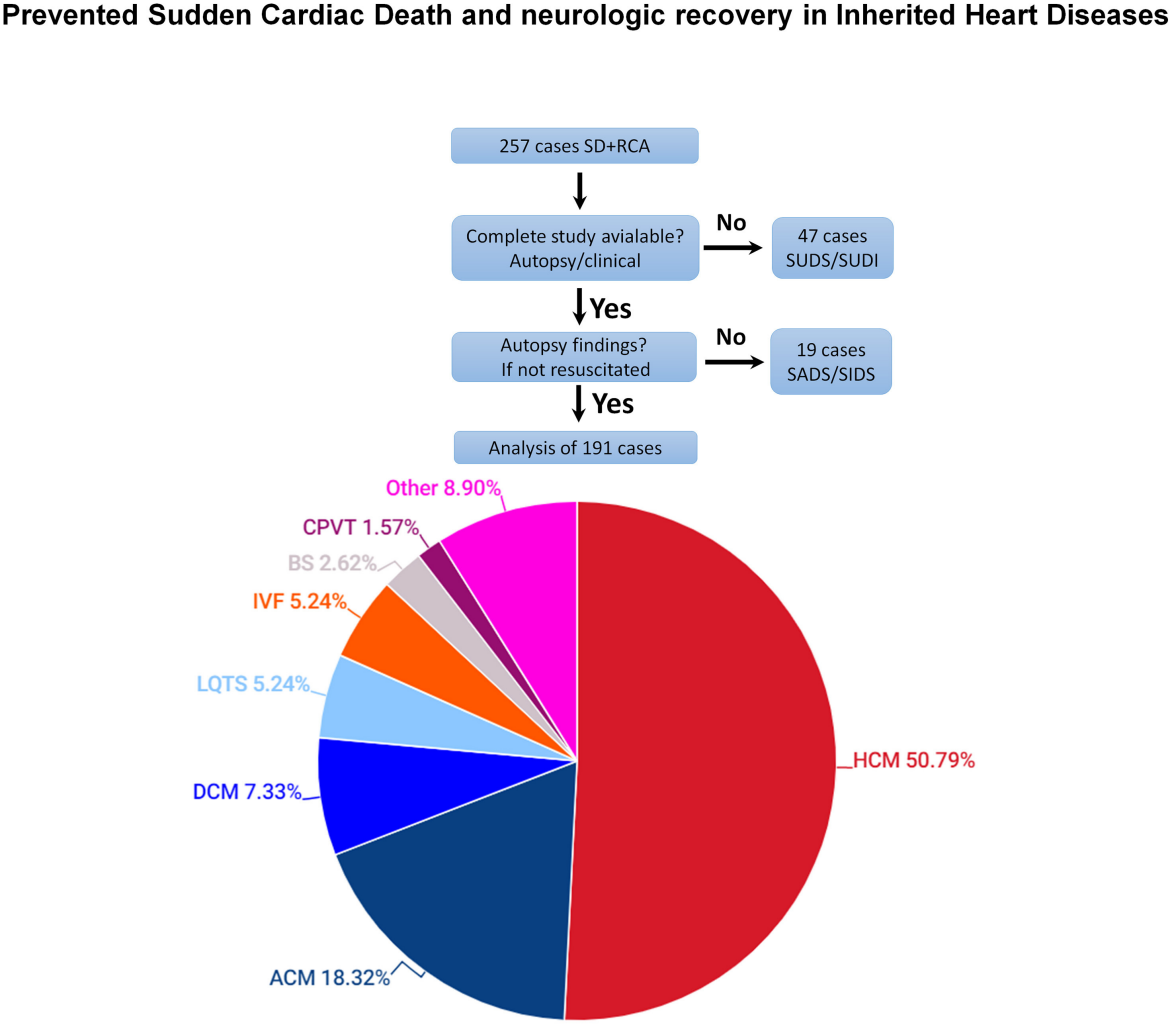
Final diagnoses of cases of resuscitated and non-resuscitated SD included in the study (autopsy or clinical study). Hypertrophic cardiomyopathy (HCM): includes 80 cases with HCM and 17 with idiopathic left ventricular hypertrophy. Arrhythmogenic cardiomyopathy (ACM): includes 24 cases with right ventricle and 11 with left ventricle involvement.

There were 19 (12.1%) cases with a negative autopsy without confirmation of channelopathy (SADS), of which seven were breast-fed babies classified as SIDS (Figure 2).

Overall, including the 191 cases of SD or RCA for which there was a complete autopsy or a cardiological study, a cardiomyopathy was diagnosed in 149 (78.0%) cases and a channelopathy was confirmed in 42 (22.0%) cases.

**Figure 3:**
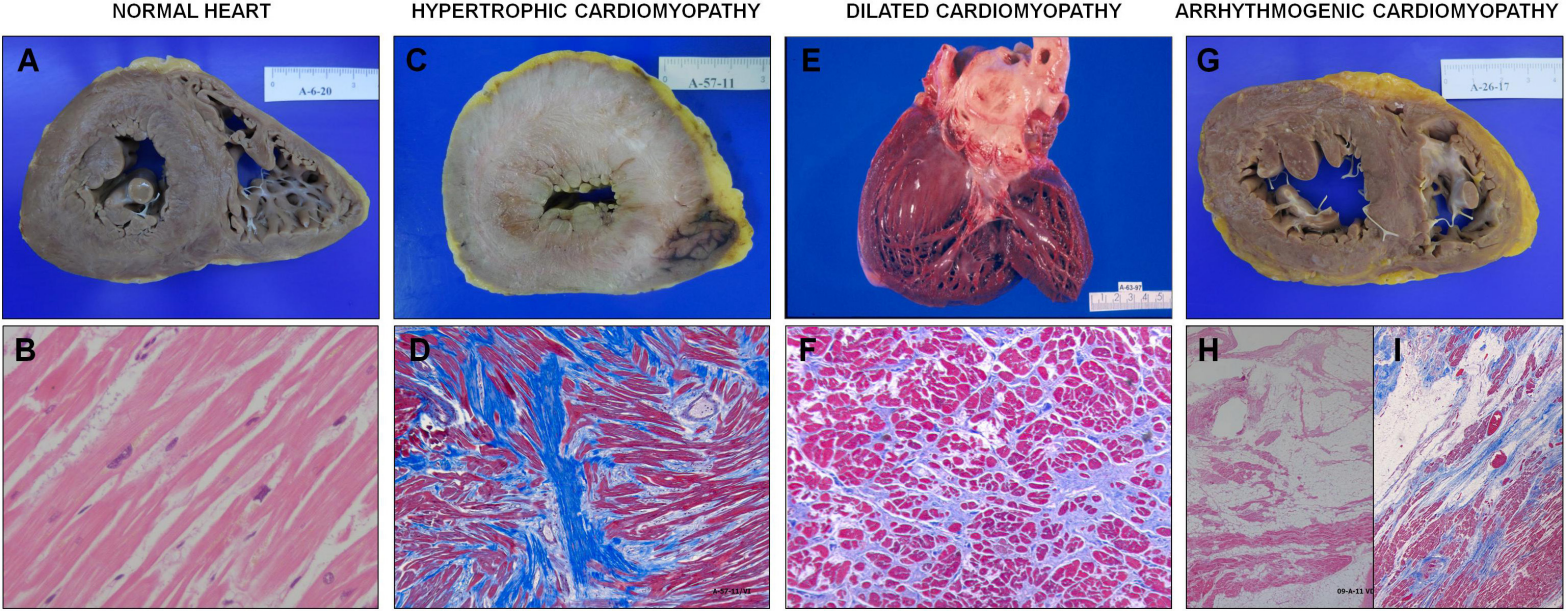
Histology of the different causes of sudden death. **(A)** and **(B)**: respectively, macro and microscopy (with hematoxylin and eosin stain) of a morphologically normal heart. **(A)** 3-mm-thick right ventricular wall, 12-mm-thick left ventricular wall; **(B)** bundles of striated muscle can be seen perfectly in line with sarcoplasms with their normal transverse striations and normochromatic oval nuclei (14-year-old female who died during an argument, diagnosed with catecholaminergic polymorphic ventricular tachycardia). **(C)** and **(D)** respectively, macro and microscopy (with Masson's trichrome stain) of hypertrophic cardiomyopathy. Fibrosis and disarray can be seen (40-year-old male who died at work). **(E)** Macroscopic appearance of dilated cardiomyopathy. The rounding appearance of the apex stands out. **(F)** Masson's trichrome stain of a heart with dilated cardiomyopathy. Patchy interstitial fibrosis surrounding muscle fibers (45-year-old male who died at home). **(G)** Section of a heart with large fatty infiltration, particularly at the right ventricle, in an arrhythmogenic cardiomyopathy. **(H)** microscopically, a massive fatty infiltration can be seen in the right ventricular wall. **(I)** subtotal fibro-fatty infiltration in the left ventricle, it extends more intensely from the epicardium to the endocardium (39-year-old male who died in his sleep).

The most common diagnosis was Hypertrophic cardiomyopathy (*n* = 80) or idiopathic left ventricular hypertrophy (*n* = 17) (97, 50.8%), arrhythmogenic right (*n* = 24) or left (*n* = 11) ventricular cardiomyopathy (35, 18.3%), dilated cardiomyopathy 14 (7.3%). Long QT syndrome was diagnosed in 10 (5.2%), idiopathic ventricular fibrillation in another 10 (5.2%), Brugada syndrome in 5 (2.6%), catecholaminergic polymorphic ventricular tachycardia in 3 (1.6%) (Table 1). Other inherited cardiac conditions consisted of 17 (8.9%) cases, including 13 mixed or unclassified cardiomyopathies, two left ventricular non-compaction, one short QT syndrome and one early repolarization syndrome. Figure 3 shows representative histological images of the different causes of sudden death.

**Table 1:** General characteristics of the study cohort regarding diagnosis.

		HCM^*^	**ACM**	**DCM**	**LQTS**	**IVF**	**BS**	**CPVT**	**Other**
	*n* (%)	90 (50.8%)	35 (18.3%)	14 (7.3%)	10 (5.2%)	10 (5.2%)	5 (2.6%)	3 (1.6%)	17 (8.9%)
Sex	Male	82 (91.1%)	26 (74.3%)	11 (78.6%)	5 (50.0%)	9 (90.0%)	4 (80.0%)	1 (33.3%)	9 (52.9%)
	Female	8 (9.9%)	9 (25.7%)	3 (21.4%)	5 (50.0%)	1 (10.0%)	1 (20.0%)	2 (66.6%)	8 (47.1%)
Age		46.3 ± 15.1	39.9 ± 12.0	53.2 ± 21.4	37.4 ± 21.8	37.3 ± 23.4	41.8 ± 14.3	44.3 ± 26.4	40.2 ± 17.5

* *Hypertrophic cardiomyopathy (HCM): includes 80 cases with HCM and 17 with idiopathic left ventricular hypertrophy. Arrhythmogenic cardiomyopathy (ACM): includes 24 cases with right ventricle and 11 with left ventricle involvement*.

In 113 (43.9%) cases, a genetic study was performed, which was positive (likely pathogenic or definitive) for 52 (46.0%) of them (33 (63.5%) known and 19 (36.5%) novel variants). Pathogenicity of the novel variants was supported by radical changes (*n* = 11) and/or cosegregation in informative families (*n* = 14). Variants of unknown significance were evidenced in 33 (42.9%) cases. In 68 (60.2%) a large high-throughput panel was used (mean 147.3 ± 83.1 genes/case). In 66 (27.7%) cases, there was no available DNA sample, in 62 (24.1%), no family consent could be obtained and in 16 (6.1%) the study was not performed for other reasons.

A total of 909 relatives from 136 (52.9%) families were clinically evaluated (mean 6.0 ± 6.7 cases per family). Familial disease was evidenced in 50 (36.8%), possible in 36 (26.4%) and negative in 50 (36.8%) families. Cardiac examinations led to diagnosis of inherited cardiac disease in 104 (11.4%) and possibly affected in 71 (7.8%) living relatives.

### Context in Which Sudden Death Occurred

Most SD or RCA episodes occurred at rest. 62 (24.1%) during daily physical activity, 60 (23.3%) while sleeping, 41 (15.9%) while resting, 24 (9.3%) during intense physical activity and 2 (0.8%) in connection with an intense emotional reaction. In 54 (21.0%) cases, the context was unknown.

### Variables Associated With Resuscitated Sudden Death

53 (20.6%) of all cardiac arrests were resuscitated. A statistically significant association was observed between SD and the male vs. female sex (83.3 vs. 16.7%, *p* = 0.002) (Table 2), and between RCA and channelopathies vs. cardiomyopathies (52.1 vs. 13.4%, *p* < 0.001) (Supplementary Table 1).

**Table 2:** General characteristics of Sudden Death (SD) and Resuscitated Cardiac Arrest (RCA) cases regarding age, sex and context of death.

		**Total**	**SD**	**RCA**	***p***
		**(*n* = 257)**	**(*n* = 204)**	**(*n* = 53)**	
Sex	Male	204 (79.4%)	170 (83.3%)	34 (64.2%)	0.002
	Female	53 (20.6%)	34 (16.7%)	19 (35.8%)	
Age		41.9 ± 18.3	42.3 ± 16.4	40.2 ± 24.2	0.556
Context	Rest/sleeping	101 (53.4%)	80 (55.6%)	21 (46.7%)	0.297
	Active	88 (46.6%)	64 (44.4%)	24 (53.3%)	
Sport	Athlete	68 (38.2%)	53 (41.1%)	15 (30.6%)	0.199
	Non-Athlete	110 (61.8%)	76 (58.9%)	34 (69.4%)	

The age of the deceased who died suddenly and were diagnosed with a channelopathy was significantly younger than the age of those diagnosed with a cardiomyopathy (31.1 ± 21.5 vs. 45.4 ± 15.3 years, *p* < 0.001).

### Prevented Sudden Death

In order to explore trends in prevented SD, an additional analysis was performed with inclusion of ICD therapies occurred in patients with inherited cardiac disease in the Region during the study period. Of the 317 cases of SD equivalent (SD, RCA or ICD therapy) there were 113 (35.6%) prevented deaths (53 (46.9%) RCA and 60 (53.1%) ICD therapies). No evidence of an increase in the proportion of prevented SD cases was observed during the study period (Supplementary Figure 1 and Supplementary Table 2).

### Neurologic Recovery After Resuscitated Sudden Death

Of the 53 cases of RCA, 15 (28.3%) died due to the brain damage caused by the cardiac arrest and 38 (71.7%) survived. 32 (60.4%) recovered a good neurological state, 4 (7.5%) had moderate impairment and 2 (3.8%) went into a coma or vegetative state (Table 3).

**Table 3:** General patient's characteristics and association with resuscitated sudden death depending of their neurologic condition after cardiac arrest.

		**Neurologic damage**			
		**Total**	**Good neurologic recovery**	**Neurologic sequelae**	***p***
		**(*n* = 53)**	**(*n* = 32)**	**(*n* = 21)**	
**Variables**					
Sex	Male	3 (64.2%)	21 (65.6%)	13 (61.9%)	0.782
	Female	1 (35.8%)	11 (34.4%)	8 (38.1%)	
Age		39.9 ± 24.3	48.1 ± 19.7	28 ± 25.9	0.003
Disease	Cardiomyopathy	20 (44.4%)	16 (51.6%)	4 (28.6%)	0.150
	Channelopathy	25 (55.6%)	15(48.4%)	10 (71.4%)	
Context	Sleeping/resting	21 (46.7%)	9 (36%)	12 (60%)	0.109
	Active	24 (53.3%)	16 (64%)	8 (40%)	
Sport	Athlete	15 (30.6%)	9 (29%)	6 (33.3%)	0.753
	Non-athlete	34 (69.4%)	22 (71%)	12 (66.7%)	
Therapeutic hypothermia	Yes	27 (50.9%)	19 (58.1%)	8 (38.1%)	0.158
	No	26 (49.1%)	13 (41.9%)	13 (61.9%)	

*Quantitative variables are expressed as mean ± standard deviation (SD) while the qualitative variables are shown as n (percentage)*.

The individuals who had a good neurological recovery were older than those who experienced moderate or severe sequelae (47.6 ± 18.4 vs. 24.7 ± 27.9 years, *p* < 0.001). After excluding SIDS cases, this difference did not reach significance (Supplementary Table 2). There was a relationship between the variables resuscitation time (min) to effective stable rhythm (direct), and recovery of consciousness achieved at hospital arrival (reverse), with severe neurological damage. Although there was a lower percentage of patients who underwent therapeutic hypothermia in the group of severe neurological impairment, this difference was not significant (8, 38.1 vs. 19, 58.1%, *p* = 0.158). Multivariate analysis of clinical predictors of severe neurological damage showed that the only associated variable was the resuscitation duration (min) (HR 1.15–IC 95%: 1.01–1.30–*p* = 0.028). No association with the type of heart disease or sex was observed.

## Discussion

This study allowed us to establish the annual rate of non-ischemic SD in our region in 1.95 cases in every 100,000 inhabitants, which means a mean of 28.6 cases per year. There are multiple series on the global incidence of SD (including ischemic), with very different results (30–160 cases/100,000) (13–15), and on its incidence among subpopulations such as athletes and young people (1–13 cases/100,000) (16–19), but the information is scarce in cases with a confirmed or suspected diagnosis of inherited heart disease (9).

The most prevalent conditions identified in our series are in line with those published by Jiménez-Jáimez et al. (20) in their study of 56 cases, on the clinical and genetic diagnosis of non-ischemic SD. In our study, with 257 cases of SD/RCA, the most frequently identified condition was hypertrophic cardiomyopathy (31.1%) followed by arrhythmogenic cardiomyopathy (13.6%). Channelopathies are overall an important cause of SD in our environment (18.9%), with idiopathic ventricular fibrillation, long QT syndrome and Brugada syndrome being the most frequent diagnosis (3, 6, 9).

The percentage of cases for which no cause could be identified after a full autopsy was 12.1%. These data are in line with those published in the literature, which range between 7 and 43% (3, 21). On the other hand, the percentage of cases for which there was no autopsy or the report was not available was relatively high (23.0%). This figure is not always available in published series, although there are references with strikingly high percentages such as the Danish series where 25% of SD in individuals younger than 35 years old had no autopsy (16).

The importance of performing a full autopsy, including a genetic study, lies in knowing the causes of SD, so that strategies toward its prevention can be designed and the living relatives at risk can be diagnosed. In a recent document, the Association for European Cardiovascular Pathology established both the procedure to perform a full autopsy from a histopathological analysis point of view, and the genetic and family study in the event that there is suspicion of an inherited heart disease (9). There are documents which offer recommendations for the study of familial heart diseases that include the performance of a thorough examination of the deceased and the collection of samples for a genetic study (11).

The 20.6% figure of patients with RCA, even though insufficient, is relatively high compared with the previous series of out-of-hospital cardiac arrest in non-cardioprotective environments (~7–10%) (4, 5, 21). Regarding cardioprotection, it is important to highlight that, at the beginning of this study in 2009, the implantation of semi-automated external defibrillators (AEDs) in the Region of Murcia was scarce and it continued to be insufficient at the end of the study period. In 2016, there were 18 AEDs/100,000 inhabitants registered, when the recommended figure is 100/100,000. The implementation of a cardioprotection program including the training of population and equipping public places with AEDs has proven to increase the resuscitation and neurologic recovery rate (5, 14, 21, 22).

The concept of prevented SD is original from our study. It is based on the premise that, with the implementation of early diagnostic measures (family screening) and the incorporation of prognostic stratification tools, it should be possible to anticipate SD. The percentage of prevented SD, which includes the cases of RCA and the ICD therapies during the study period, was established in 35.6%. Although this percentage is relevant, we failed to observe a significant increase in prevented SD during the study period (Supplementary Figure 1). It is important to note that most risk scores have been published during the past few years; particularly for hypertrophic cardiomyopathy in 2014 (23), and for arrhythmogenic right ventricular cardiomyopathy in 2018 (24), which are the two most frequent diseases among the cases of SD in our series. Risk stratification is still controversial in other conditions such as Brugada syndrome, dilated cardiomyopathy or arrhythmogenic left ventricular cardiomyopathy (25, 26).

The main problem after RCA is not maintaining heart function, but preventing neurologic consequences, as much as possible. It is well-known that the earlier basic and advanced resuscitation is performed, the greater the extent of neurologic recovery is (22). This is related to the context and the identification of cardiac arrest. In our series, the percentage of patients with good neurologic recovery was 60.4%. We identified two variables associated with successful resuscitation: a channelopathy, in contrast with a cardiomyopathy, and female sex; and a variable associated with neurologic recovery: resuscitation time (min) until restoring effective heart rhythm.

Therapeutic hypothermia was indicated for 52% of patients with RCA. Despite the fact that this treatment has proven to be effective for neuronal preservation in postanoxic encephalopathy (27), we did not observe an association with neurologic consequences in our series. It is true however that an important percentage of patients for whom it was not indicated had recovered consciousness (35%) after cardioversion and therefore had a good prognosis.

## Limitations

This study has the limitations inherent to a partially retrospective study. The number of cases is insufficient for an in-depth analysis of the predictors for successful resuscitation. Although the collection of cases of SD was prospective and there was a collaboration agreement between the Institute for Legal Medicine and the Regional Ministry of Health, an autopsy was not performed or the report was not available in 23% of cases (aged 42.3 ± 19.3 years, 72.3/27.7% male/female). In those cases, the assumption that there was an underlying inherited heart disease was based on the personal history or the family study (youth or children, absence of cardiovascular risk factors, previous examinations, family study showing evidence of cardiomyopathy or channelopathy). There was a selection of patients who underwent therapeutic hypothermia after cardiac arrest with a bias toward the inclusion of the most severe patients. This treatment was not available in our Region for cases of RCA before 2012. The scale of neurologic damage has been specifically designed for this study, as simple scales which allowed for a retrospective classification were not available in the literature.

## Conclusions

One in every five non-ischemic cardiac arrests is successfully resuscitated. Male sex is predominant in SD cases. Women and channelopathies are more prevalent among resuscitated cases. Prevented SD occurs in one in every three patients with a probable or definitive diagnosis of familial heart disease. In this setting about two in every three resuscitated patients have a good neurologic recovery.

## Data Availability Statement

The raw data supporting the conclusions of this article will be made available by the authors, without undue reservation.

## Ethics Statement

The studies involving human participants were reviewed and approved by Human research committee from University Hospital Virgen Arrixaca. The patients/participants provided their written informed consent to participate in this study.

## Author Contributions

JG, JH, and FP: conceptualization. AR, JS, MN, and EN: methodology. MO and DL: software. JG and MS-M: validation. JG, JS, CM, and HG: formal analysis. HG, MS-M, and CG: investigation. MN, JS, CM, JH, and MO: data curation. JG, MS-M, and AR: writing—original draft preparation. JG: funding acquisition. All authors have read and agreed to the published version of the manuscript.

## Conflict of Interest

The authors declare that the research was conducted in the absence of any commercial or financial relationships that could be construed as a potential conflict of interest.

## Supplementary Material

Supplementary Figure 1 Number of cases of Sudden Death, Resuscitated Cardiac Arrest and ICD therapy during the study period. The dotted line represents the proportion of prevented Sudden Death (prevented SD = RCA + ICD therapy/SD + RCA + ICD therapy).

Supplementary Table 1 General characteristics of the cohort regarding the diagnosis of Cardiomyopathy or Channelopathy.

Supplementary Table 2 Prevented sudden death during the study period (years): includes sudden death, resuscitated cardiac arrest and ICD therapies.
