# Supplementary figures and images for: Prevented Sudden Cardiac Death and Neurologic Recovery in Inherited Heart Diseases
Source: Front Cardiovasc Med. 2021 Mar 15;8:634300. doi: 10.3389/fcvm.2021.634300 (PMC8005516; doi:10.3389/fcvm.2021.634300)

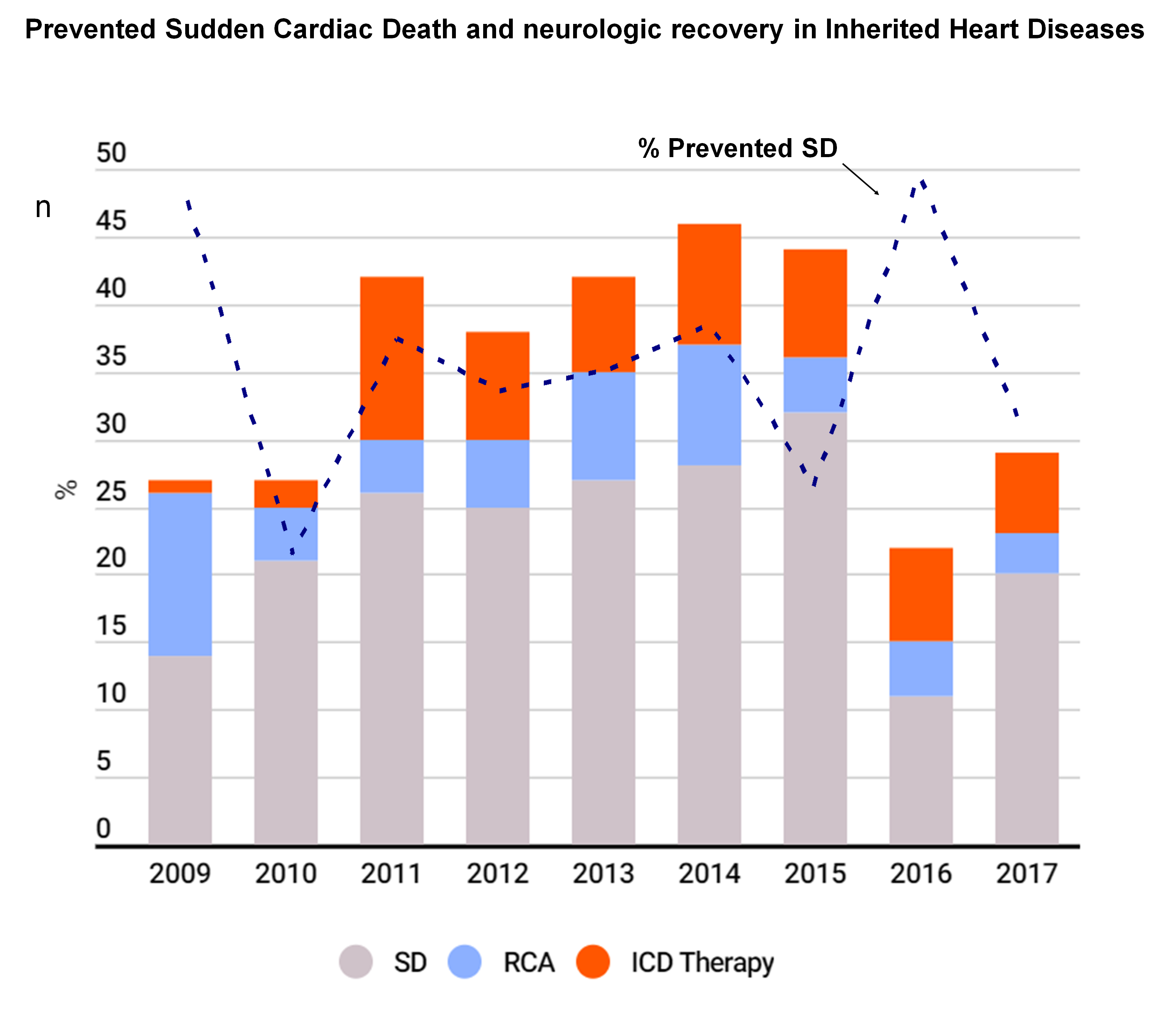

Supplement: Supplementary Figure 1 — Number of cases of Sudden Death, Resuscitated Cardiac Arrest and ICD therapy during the study period. The dotted line represents the proportion of prevented Sudden Death (prevented SD = RCA + ICD therapy/SD + RCA + ICD therapy). [file Image_1.TIF]
