# Supplementary material for: Prevented Sudden Cardiac Death and Neurologic Recovery in Inherited Heart Diseases
Source: Front Cardiovasc Med. 2021 Mar 15;8:634300. doi: 10.3389/fcvm.2021.634300 (PMC8005516; doi:10.3389/fcvm.2021.634300)
Supplement: Supplementary Table 1 — General characteristics of the cohort regarding the diagnosis of Cardiomyopathy or Channelopathy. [file Data_Sheet_1.PDF]

Table 1 supplementary. General characteristics of the cohort regarding the diagnosis of Cardiomyopathy or Channelopathy.

		**Total** (n = 197)	**Cardiomyopathy** (n = 149)	**Channelopathy** (n = 48)	**p**
**Sex**	Male	162 (82.2%)	129 (86.6%)	33 (68.8%)	0.005
	Female	35 (17.8%)	20 (13.4%)	15 (31.3%)	
**Age**		41.9±18	45.4±15.3	31.1±21.5	<0.001
**Status**	Sudden Death	152 (77.2%)	129 (86.6%)	23 (47.9%)	<0.001
	Resus. Cardiac Arrest	45 (22.8%)	20 (13.4%)	25 (52.1%)	
**Context**	Rest/sleeping	77 (51.3%)	53 (49.1%)	24 (57.1%)	0.375
	Active	73 (48.7%)	55 (50.9%)	18 (42.9%)	
**Sport**	Athlete	57 (38.8%)	44 (42.3%)	13 (30.2%)	0.172
	Non-athlete	90 (61.2%)	60 (57.7%)	30 (69.8%)	
