# Supplementary material for: Prevented Sudden Cardiac Death and Neurologic Recovery in Inherited Heart Diseases
Source: Front Cardiovasc Med. 2021 Mar 15;8:634300. doi: 10.3389/fcvm.2021.634300 (PMC8005516; doi:10.3389/fcvm.2021.634300)
Supplement: Supplementary Table 2 — Prevented sudden death during the study period (years): includes sudden death, resuscitated cardiac arrest and ICD therapies. [file Data_Sheet_2.PDF]

Table 2 supplementary. Prevented sudden death during the study period (years): includes sudden death, resuscitated cardiac arrest and ICD therapies.

		Prevented SD (n =113)	
	**SD (non-resuscitated)** (n = 204)	**Resus. Cardiac Arrest** (n =53)	**ICD Therapy** (n = 60)
**2009**	14 (51.9%)	12 (44.4%)	1 (3.7%)
**2010**	21 (77.8%)	4 (14.8%)	2 (7.4%)
**2011**	26 (61.9%)	4 (9.5%)	12 (28.6%)
**2012**	25 (65.8%)	5 (13.1%)	8 (21.1%)
**2013**	27 (64.3%)	8 (19%)	7 (16.7%)
**2014**	28 (60.8%)	9 (19.6%)	9 (19.6%)
**2015**	32 (72.7%)	4 (9.1%)	8 (18.2%)
**2016**	11 (50%)	4 (18.2%)	7 (31.8%)
**2017**	20 (69%)	3 (10.3%)	6 (20.7%)
**Total**	204 (64.4%)	53 (16.7%)	60 (18.9%)

Prevented sudden death is defined as the sum of resuscitated cardiac arrest and ICD therapy. Numbers and proportion of prevented sudden death is represented in figure 4.
